# Common School Convention

**Published:** 1846-10

**Authors:** 


### PART IV.—EDITORIALS.

### ARTICLE VII.

### COMMON SCHOOL CONVENTION.

In June, 1845, a State Common School Convention was held at Jacksonville, Ill., upon the adjournment of which, it was resolved: "that another should be held at Chicago in the autumn of 1846, immediately succeeding the Agricultural and Mechanical Exhibition then and there to be held." Messrs Geo. W. Meeker, William Jones and Wm. H. Brown, were appointed a committee to make the arrangements, in accordance with the resolution.

We have before us the circular issued by this committee calling upon all the friends of education in the West, to attend and aid to make the meeting profitable and interesting, and stating the objects of the convention and the subjects which it is intended will be discussed. An extract from the circular will give our readers an idea of the objects of the movement.

"The doings of the Convention, or the measures they may adopt, it is, of course impossible for us to announce. We can state however, that the following subjects were selected by the Convention at Jacksonville, and the following persons appointed to prepare essays upon them, which will be read at the Convention at Chicago.

"1. How may a system of Education be so conducted as to afford the best preparation for the various professional, agricultural, mechanical and commercial pursuits? Upon what principles, and to what extent should the course of instruction be accommodated to each class?

"2. Should the same system of education be pursued in regard to males and females, or upon what principle and to what extent should any difference be made?

"3. How can we extend the means of instruction to all classes of community?

"4. How may we accomplish the moral culture and elevation of all connected with our schools?

"5. Can a system of common schools be made efficient without the division of the state into political townships?

"6. Should the communication of knowledge, or the discipline of mind, be the primary object of the teacher? What course and methods of instruction are best adapted to these ends?

"7. How can we best elevate the character and qualifications of teachers?

"8. Are colleges and female seminaries indispensable as part of a system of general education?

"9. Should the course of instruction in colleges and female seminaries be conducted with reference to the preparation of competent teachers, and should legislative aid be granted, at least to the extent of the University fund for the attainment of this end? The nine queries were assigned as follows:

No. 1. J. M. Sturtevant, President of Illinois College.
2. William H. Williams, Jacksonville.
3. Francis Springer, Springfield.
4. Prof. J. B. Turner, Illinois College.
5. A. W. Henderson, Chicago.
6. Rev. C. E. Blood, Madison County.
7. John S. Wright, Cook County.
8. Hon. Wm. Brown, Jacksonville.
9. T. M. Post, do.

If either of the individuals appointed shall be unable to be present, he will be expected to prepare his essay and forward to one of the undersigned, that it may be read. The essays will furnish the basis for discussing the subjects upon which they treat, and if any one should be unable to prepare his essay, it is expected he will procure some one to take his place capable of discharging the duty. The opening address will be delivered by Wm. H. Brown, Esq.

"To teachers, we would say, it is in contemplation to organize a Teachers' Institute immediately succeeding the Convention, convening probably on Monday morning, and continuing from one to two weeks. At the East, they have been tried for the last few years with the most happy results. The plan pursued, is to organize a regular school of the teachers, the most experienced alternately being appointed to instruct the others. Methods of imparting instruction in the various branches are presented, together with plans of government, discipline, &c. The benefits to teachers, of thus meeting together to learn the improvements in the art, profiting by each other's experience, and of increasing their interest and pride in their high calling, must be apparent to the most casual observer. It is expected that this Western Institute, will number several hundred members, all of whom will be gladly entertained by our citizens without charge; and when they return home, they will be prepared to organize county and neighborhood Institutes all over the West. No teacher who appreciates the importance and dignity of his vocation, will refuse to spend two or three weeks in going to and attending upon such a meeting; and if the parents realize the value of good instruction, they will not refuse to be at the little expense of the journey, which will be repaid with a thousand fold profit to their children. No teacher will regret his attendance, but it is to be feared very many of them will regret not having made sufficient effort to be present.

Teachers, too—those who know from experience the wants of the West as to education—are the ones of all others who are needed to make the proceedings of the Convention preceding the Institute practical and profitable."

The citizens of Chicago, at a meeting held on the 16th of July, appointed a large committee of arrangements, to make preparations for the entertainment of delegates, &c. &c. The following were the resolutions adopted on that occasion:

"*Resolved*, That we congratulate our fellow citizens in having our city selected for a Western Educational Convention to be held in Oct., and that we pledge ourselves to use all reasonable exertions to make the meetings and visits of the members among us, both pleasant and profitable.

"*Resolved*, That we most cordially tender the hospitalities of Chicago to all who will attend, and also to those who may remain to aid in the formation of a Teachers' Institute.

"*Resolved*, That the following persons be appointed a Committee to see that each member of the Convention is provided with a place of abode; that as they arrive in the city, they be requested to record their names at the Prairie Farmer Office, where some one will be in waiting to direct them to their lodgings, and that those who attend be requested to send their names at an early day to Geo. W. Meeker, Esq., that a probable estimate may be made of the number that may be expected."

It is expected that the Convention will be addressed by a number of gentlemen interested in the subject, as will be seen from the following:

"It may increase the desire with some to state that HENRY BARNARD, Superintendent of schools in Connecticut and Rhode Island, and Prof. DEWEY of Rochester, N. Y., are expected to be present; and that it is possible HORACE MANN, superintendent of Schools in Massachusetts, and other distinguished friends of Education from abroad may also attend. No efforts will be spared to bring together as many experienced and energetic friends of the cause as can make it within their power to attend on that occasion."

We hope that our readers will exert their influence in procuring a large attendance, as our profession as well as other classes of the community, is deeply interested in the cause of General Education. From no one source are we to anticipate more hopeful influence in the abolition of empiricism, and a proper appreciation of true medical science, than from the general enlightenment of the public mind. Thursday evening, October 8th, is appointed for the opening ceremonies of the Convention.

